# Evaluation of *Pasteurella multocida* serotype B:2 resistance to immune serum and complement system 

**Published:** 2017-09-15

**Authors:** Saeed Ataei Kachooei, Mohammad Mehdi Ranjbar, Saba Ataei Kachooei

**Affiliations:** 1 Department of Bacterial Poultry Diseases, Razi Vaccine and Serum Research Institute, Agricultural Research, Education and Extension Organization (AREEO), Karaj, Iran;; 2 Department of Animal Virology, Razi Vaccine and Serum Research Institute, Agricultural Research, Education and Extension Organization (AREEO), Karaj, Iran;; 3 Department of Life Sciences, Faculty of Life Sciences, Kharazmi University, Karaj, Iran.

**Keywords:** Cattle, Complement, Hemorrhagic septicemia, *Pasteurella*

## Abstract

Members of gram-negative bacteria family* Pasteurellaceae*, include a large number of important economically human and veterinary pathogens. Organisms belonging to the family can colonize in mucosal surfaces of the respiratory, alimentary, genital tracts and cause diseases in various mammals, birds, and reptiles. Hemorrhagic septicemia is an acute disease of cattle and buffaloes in tropical countries caused by *Pasteurella multocida* serotype B:2. In the present study, the possible bactericidal activity of immune calf sera in the presence and absence of complement system was investigated. The results showed that *P. multocida* B:2 is highly resistant to positive serum, containing high levels of IgG and IgM obtained from calves after vaccination, and complement activity in normal fresh calf serum. This organism also grew rapidly in the normal fresh calf serum and the mixture of positive serum as well as normal fresh calf serum. As a control test an *E. coli* strain was subjected to the same experiment and found completely sensitive to the bactericidal activity of complement in calf and guinea pig fresh sera. Results were indicative of the presence of inhibitory mechanism(s) in* P. multocida* B:2 against bactericidal activity of immune calf serum and complement system.

## Introduction

*Pasteurella multocida*, a gram-negative, nonmotile, coccobacillus, has been recognized as an important veterinary pathogen for over 130 years. Based on capsule antigens, *P. multocida* is classified into five serogroups as A (hyaluronic acid), B, D (heparin), E and F (chondroitin) and according to lipopolysaccharide (LPS) antigens is classified into 16 serotypes.^1^^,^^2^ Specific serotypes of *P. multocida* are associated with different diseases in cattle, buffalo, sheep, goats, camels, pigs, poultry and other animals.^1^^,^^3^^,^^4^ The organism has acquired the ability of surviving and growth on mucosal surfaces of respiratory, urinary, digestives tracts and consequently caused wide range of different diseases most importantly hemorrhagic septicemia (HS) of cattle and buffalo, fowl cholera (FC) of chicken, and atrophic rhinitis of pig. In some septicemia causing serotypes such as HS and FC the organism is able to survive the destructive effects of serum.^5^

The destructive effects of fresh serum on gram-negative bacteria is assumed to be one of the consequences of the activity of host complement system.^6^ In *P. multocida* serotype A, the causative agent of fowl cholera, it has been shown that a virulent strain was resistant to turkey serum while an avirulent strain was serum-susceptible.^7^^,^^8^ Similar results have been reported by other studies and serum resistance has been considered as a tool to assess the virulence in FC-associated strains.^9^ However, lesions have also been reported in chickens infected by serum-sensitive strains^10^ and the relationship between virulence and serum resistance is complicated in the chicken. ^11^ The exact molecular mechanisms of the innate and adaptive immune cell responses are not fully understood for any of this virulence factors.^12^

*Pasteurella multocida* bears a capsule and LPS which play major role in its cell surface.^13^ These polysaccharidic components are widely used for bacterial classification, responsible for non-immunological and immunological interactions of bacteria with hosts and also involved in the avoidance of host innate immune mechanisms-such as resistance to phagocytosis, complement-mediated killing, and the bactericidal activity of antimicrobial peptides.^13^

Different monoclonal antibodies reacting with the LPS of the bacterium could opsonise *P. multocida* for phagocytosis by mouse macrophages, but were not bactericidal in the presence of complement.^14^^,^^15^ Experiments on sensitivity of *P. multocida* B:2 to naive calf serum have shown that the organism is quite resistant to complement-mediated killing. This study showed that both the wild type and an acapsular mutant grew rapidly in either fresh or heat-treated calf serum. It was concluded that capsule does not play any role in resistance to complement. Similar results were obtained for growth in mouse serum.^16^

At the end of the 19^th^ century, the complement system was discovered as a heat-labile component of serum that augmented or complemented its bactericidal activity.^17^ This system is composed of about 16 plasma proteins which interact with each other or with other cellular components of immune system such as phagocytes and T cells.^18^ Complement, along with other plasma derived pattern recognition receptors (PRRs), coats the microbes (opsonization), allowing recognition and binding by opsonic receptors on host phagocytes.^19^ The complement system can be activated through three different pathways. The classical pathway, which is triggered directly by pathogen or indirectly by antibody binding to the pathogen surface; the lectin pathway and the alternative pathway. All three pathways can be initiated independently of antibody as part of innate immunity.^20^ Finally, all three pathways culminate in the formation of the C3 and C5 convertases that, in turn, generate the anaphylatoxins C3a and C5a, the membrane-attack complex (MAC; C5b-C9) and the opsonin C3b.^20^ Activation of the complement system in response to extracellular bacteria may be through the alternative pathway when the opsonin C3b binds to the surface of the microbe.^21^ The complement system can also affect T-cell responses through the direct modulation of T-cells or indirectly through the alteration of immunomodulatory cells, particularly antigen-presenting cells (APCs).^18^

Regulation of complement system is extremely required for protection of host cells and tissue from destructive effects of complement elements.^17^ To prevent uncontrolled activation of complement system a family of regulators has evolved in host immune system to defend host cells from opsonization or lysis. The C3 and C5 convertases enzyme activity in plasma are heavily controlled by two plasma inhibitors called factor H (FH) destroy the convertase enzymes of the alternative pathway and C4 binding protein (C4BP) performs the same activity in the classical pathway.^17^

In the present study it was aimed to evaluate the resistance of *P. multocida* serotype B:2 to the bactericidal activity of immune serum and complement system by serological methods. The resistance of *P. multocida* to the destructive effect of fresh serum was also compared to an *E.coli* lab strain as a candidate of non-pathogenic gram-negative bacterium.

## Materials and Methods

The serum sensitivity assay was done according to Boyce and Adler with modifications.^16^ Guinea pig complement serum (Sigma, St. Louis, USA) and sera from young calves (fresh normal calf serum) were used as the complement source. Blood was obtained from normal young calves in sterile glass containers when the calves were put down at the Department of Pathology, School of Veterinary Medicine, University of Glasgow, Scotland.

**Fig. 1:**
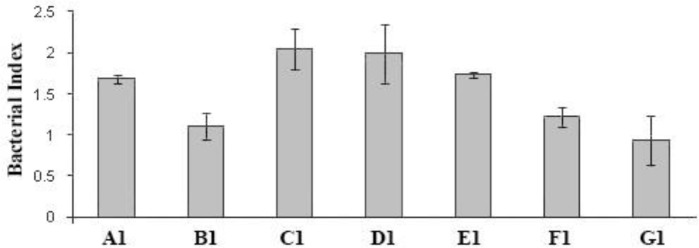
Bactericidal activity of bovine serum on *P. multocida. *Data were the average of two assays ± standard deviation. An index of < 1 would indicate a bactericidal effect. The bactericidal index was defined as CFU mL^-1^ after incubation for 1 hr divided by CFU mL^-1^ at time zero. All three groups C1, D1, and E1 showed roughly the same growth rate and there was no significant difference between these average growth indexes (*p *> 0.05). A1: Immune serum + normal calf serum, B1: Heated immune serum + heated normal calf serum, C1: Immune serum + PBS, D1: Heated immune serum + PBS, E1: Normal calf serum + PBS, F1: Heated normal calf serum + PBS, and G1: PBS. Each well of a 96-well microtitre plate contained 25 μL of immune serum from a calf with high level of IgG and IgM antibody (Immune.Ser), with or without 25 μL of fresh normal calf serum (NC.Ser) or 25 μL heated Immune.Ser with or without 25 μL heated NC.Ser or 25 μL fresh NC.Ser or 50 μL heated NC.Ser or 50 μL PBS. A 50 μL suspension of *P. multocida* B:2 was added to each well and then incubated for 1 hr at 37 ˚C. Sample of 10 µL from undiluted, and dilutions of 10^-1^ and 10^-2^ were taken before and after incubation and were plated out on serum bovine albumin (SBA) and then incubated overnight

**Fig. 2:**
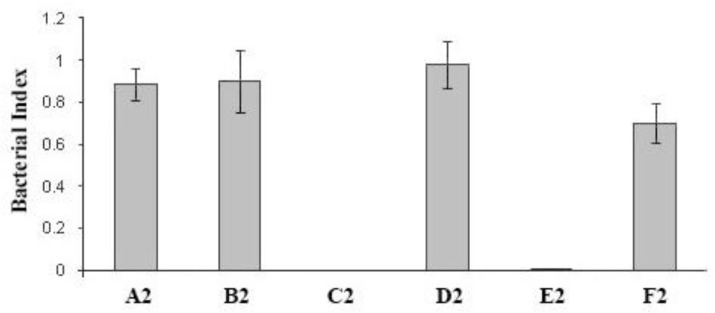
Bactericidal activity of calf serum and guinea pig complement on *E. coli.* Data were the mean of two assays ± standard deviation. An index of < 1 would indicate a bactericidal effect. There was significant difference between D2 and C2 growth indexes mean (*p* < 0.05). There was no significant difference between C2 and E2 growth indexes (*p* > 0.05). A2: Fetal calf serum, B2: Heated fetal calf serum, C2: Normal calf serum, D2: Guinea pig complement, and F2: PBS. Each well of a 96-well microplate contained 50 μL of fetal calf serum (Fetal Calf Ser), heated fetal calf serum (Heat.Fetal Calf Ser), fresh normal calf serum (NC.Ser) or heated calf serum or guinea pig complement or PBS. A 50 μL of a suspension of *E. coli* was added to each well and then incubated for 1 hr at 37 ˚C. Sample of 10 μL from undiluted, and dilutions of 10^-1^ and 10^-2^ were taken before (time zero) and after incubation and were plated out on SBA and then incubated overnight. The bactericidal index was defined as CFU mL^-1^ after incubation for 1 hr divided by CFU mL^-1^ at time zero

Collected blood was then left at room temperature for 4 hr and the serum was separated, aliquot in 2-mL capped class tubes and kept at – 20 ˚C. The assay was performed in 96-well, tissue culture-grade, flat-bottomed, micro-plates (Costar, Cambridge, USA). To prepare a bacterial suspension, a few separate colonies from an overnight culture of *P. multocida *B:2 on serum bovine albumin (SBA) were inoculated into 9 mL of brain heart Infusion (BHI) broth (Sigma) and incubated statically overnight at 37 ˚C. An inoculum of 0.5 mL was transferred into 9 mL of pre-warmed BHI broth and incubated at 37 ˚C with shaking at 200 rpm for 4 hr. The cells were collected by centrifugation at 3,000 *g* for 30 min at 4 ˚C and then washed once with phosphate-buffered saline (PBS; pH 7.2) and re-suspended in PBS. A bacterial suspension of 2 × 10^6 ^CFU mL^-1^ in PBS was prepared from *P. multocida* B:2 or *E. coli* and used as the bacterial suspension. Different test sera (25 μL) were added in duplicate in the wells of sterile micro-plates, except when evaluating the effect of complement alone, where the serum was replaced with PBS. Then, bacterial inoculums (50 μL) were added to each well. The complement source (25 μL) was added, except for the control wells for evaluation of the effect of test sera on *P. multocida* in the absence of complement, and the complement control wells in which heat-inactivated (56 ˚C for 30 min) complement was added. Plates were then covered with a lid and incubated at 37 ˚C for the required time. Diluted (10^-1^ and 10^-2^) and undiluted samples (10 μL) from each well were placed on SBA in 10 ×10 cm square plates (Bibby Sterilin Ltd., Staffordshire, UK) at the beginning (time zero) and at 1 hr after incubation. The plates were allowed to dry and then incubated overnight at 37 ˚C. The count of each countable test was calculated as the mean number of colonies derived from in each of the three different dilutions. The killing effect of each serum was calculated as a bactericidal index: CFU mL^-1^ after 1 hr incubation divided by CFU mL^-1^ at time zero. Data were presented as the average of two assays ± standard deviation in groups signed as A1 to G1 including A1: Immune serum + normal calf serum, B1: Heated immune serum + heated normal calf serum, C1: Immune serum + PBS, D1: Heated immune serum + PBS, E1: Normal calf serum + PBS, F1: Heated normal calf serum + PBS, and G1: PBS in Figure 1 and as A2 to F2 including A2: Fetal calf serum, B2: Heated fetal calf serum, C2: Normal calf serum, D2: Guinea pig complement, and F2: PBS in Figure 2. Data were processed using SPSS (version 21; SPSS Inc., Chicago, USA) and compared by *t*-test method.

Sera with high IgG and IgM titres obtained after the second booster vaccination with an experimental live attenuated vaccine on day 42 from intramuscular (IM)-vaccinated calves were selected for use in this assay. *Pasteurella multocida* wild type was incubated with these sera in the presence of fresh normal calf serum, as a source of complement, to study their bactericidal effects on the organism. A control included the assay with the same conditions in which PBS was used instead of serum. A typical result obtained with one such serum is shown in Figure 1. None of the immune sera obtained from vaccinated calves or fresh calf serum obtained from naive calves, showed any suppressive effects on growth of *P. multocida,* which grew in these sera as shown by a bactericidal index > 1, rather than indices < 1 which would represent- a killing effect. This, in contrast to results obtained with  *E. coli* strain K12 which was not killed in fetal calf serum, but was rapidly killed in fresh calf serum and guinea pig serum as complement sources. Heating (56 ˚C for 30 min, for inactivation of complement) fresh calf or guinea pig sera had no suppressive effects on growth rate of *E. coli*.

## Results

Our experiments on assessment of the bactericidal activity of the serum showed that, *P. multocida* B:2 was highly resistant to positive serum (containing high levels of IgG and IgM antibodies, obtained from immunized calves one week after second IM booster vaccination) and also highly resistant to complement activity in normal fresh calf serum (Fig. 1). The wild type strain of* P. multocida* B:2 was incubated in sera obtained from vaccinated calves with high titers of IgG and IgM, in the presence or absence of normal fresh calf serum as a source of complement, to study its sensitivity to complement-mediated killing. 

The* P. multocida* B:2 strain was resistant and grew in the positive and heated positive sera. The strain also grew in the normal fresh calf serum and the mixture of positive serum and normal fresh calf serum. Heated normal fresh calf serum and the mixture of heated positive serum and heated normal calf serum had no effect on the strain. Each of the fresh and positive sera had stimulatory effects on growth of the strain allowing the number of organisms to double during incubation. On the other hand, after heating of the sera, growth was not apparent, maybe because of the destruction of the growth stimulatory agent(s) due to heating. 

In order to check the complement activity, the bactericidal effect of the sera was also assessed on an *E. coli* K12 strain (a non-pathogenic strain mainly used for molecular biology studies in the lab). Fresh normal calf serum completely killed all the organisms within one hour, but the heated calf serum had no effect (Fig. 2). Guinea pig complement was also able to kill the *E. coli* K12 strain. Fetal calf serum (used as an additive for mammalian cell cultures) had no effect on the organism. 

## Discussion

The rapid progress of HS disease in naive animals is indicative of resistance of the bacterium to innate immunity elements such as the complement system, and resistant strains show that they are more virulent but the exact mechanism of this resistance is poorly understood.^22^ These mechanisms are evolved for adaption and survival in host and avoidance from recognition of immune system.^5^ Some of these mechanisms are related to *P. multicida* toxin by affection on bone homeostasis to evade immune system and other mechanisms which act as anti-immune strategies for instance hiding from immune-surveillance, blockage of specific immunity, down regulation of apoptosis, modifying Toll-like receptor (TLR) signaling and manipulation of intrinsic cellular pathway.^5^^,^^23^

Our results supported another study which showed that *P. multocida* B:2 was highly resistant to complement activity of normal bovine and murine serum and grew rapidly in either fresh or heat-treated calf serum.^16^ Our findings are also in agreement with studies which showed that different monoclonal antibodies reacting with the LPS of the bacterium were not bactericidal in the presence of complement, although these antibodies were able to opsonise the bacterium for phagocytosis by mouse macrophages.^14^^,^^15^ It would be predicted that, anti-LPS antibodies would be raised in calves after vaccination with the *aroA* strain. Although, it is generally believed that immunity to *P. multocida* B:2 is antibody-mediated, and the exact mechanisms involved in protection conferred by antibodies is not clear. Our results showed that *in vitro* exposure of the organism to sera with high levels of antibody in the presence and absence of complement does not have an adverse effect on the organism, since all three groups C1, D1, and E1 showed roughly the same growth rate and there was no significant difference between these mean growth indexes. There was significant difference between D2 and C2 growth indexes mean. There was no significant difference between C2 and E2 growth indexes. As also shown by our experiments, challenge of vaccinated animals, which had high levels of antibody, prevented the progress of disease. Thus, it could be assumed either these antibodies promoted clearance by opsono-phagocytosis or neutralized the activity of important virulence factor(s), but the nature of these remains elusive. It would be of interest to test the opsonising effect of these antibodies in vitro using isolated macrophages or a cell line. It should be noted that protection did not prevent the localization of the *P. multocida* serotype B:2 in tonsillar lymph nodes. This indicated that antibodies against the organism might contribute to protection by inhibiting the rapid spread of the organism through the body, a possible role of protective antibodies suggested for infection with *P. multocida* serotype A3.^24^ In addition, for HS-causing *P. multocida *B:2*,* latent carrier animals have high levels of antibody, it has also been suggested that these antibodies protect the carrier animals by inhibiting the rapid spread of the organism through the body.^25^

The mechanism of *P. multocida* resistance to destructive elements of the complement system has not been cleared. The resistance mechanism for some other pathogens such as *Neisseria meningitidis*^26^^,^^27^ and *Leptospira interrogans* ^28^^-^^31^ has been identified as binding of host regulatory complement factors of FH and C4BP to the surface of the organism. It has also been shown by a recent study on *P. pneumotropica*, an opportunistic bacterium of rodent pasteurellosis also able to cause severe infection in immunodeficient mice, and also a potential source for human contamination, that the mechanism of resistance to human complement system is through binding of FH and C4BP regulatory factors.^22^ For *P. multocida* the mechanism of resistance has not been cleared, tough similar resistance mechanism of FH and C4BP are quite expectable. In addition, other bacterial resistance mechanisms to complement activity such as activation of bacterial proteases could play the role. It is suggested that further research is needed and current findings inevitably inspire new questions on other mechanisms of survival and immune escape mechanism of *P. multicida.*
